# Weight Status Modulated Brain Regional Homogeneity in Long-Term Male Smokers

**DOI:** 10.3389/fpsyt.2022.857479

**Published:** 2022-06-06

**Authors:** Mengzhe Zhang, Xinyu Gao, Zhengui Yang, Xiaoyu Niu, Jingli Chen, Yarui Wei, Weijian Wang, Shaoqiang Han, Jingliang Cheng, Yong Zhang

**Affiliations:** ^1^Department of Magnetic Resonance Imaging, The First Affiliated Hospital of Zhengzhou University, Zhengzhou, China; ^2^Key Laboratory for Functional Magnetic Resonance Imaging and Molecular Imaging of Henan Province, Zhengzhou, China; ^3^Engineering Technology Research Center for Detection and Application of Brain Function of Henan Province, Zhengzhou, China; ^4^Engineering Research Center of Medical Imaging Intelligent Diagnosis and Treatment of Henan Province, Zhengzhou, China; ^5^Key Laboratory of Magnetic Resonance and Brain Function of Henan Province, Zhengzhou, China; ^6^Key Laboratory of Brain Function and Cognitive Magnetic Resonance Imaging of Zhengzhou, Zhengzhou, China; ^7^Key Laboratory of Imaging Intelligence Research Medicine of Henan Province, Zhengzhou, China

**Keywords:** regional homogeneity, resting-state functional magnetic resonance imaging, long-term smoking, overweight, interaction

## Abstract

**Background:**

Tobacco smoking and being overweight could lead to adverse health effects, which remain an important public health problem worldwide. Research indicates that overlapping pathophysiology may contribute to tobacco addiction and being overweight, but the neurobiological interaction mechanism between the two factors is still unclear.

**Methods:**

The current study used a mixed sample design, including the following four groups: (i) overweight long-term smokers (*n* = 24); (ii) normal-weight smokers (*n* = 28); (iii) overweight non-smokers (*n* = 19), and (iv) normal-weight non-smokers (*n* = 28), for a total of 89 male subjects. All subjects underwent resting-state functional magnetic resonance imaging (rs-fMRI). Regional homogeneity (ReHo) was used to compare internal cerebral activity among the four groups. Interaction effects between tobacco addiction and weight status on ReHo were detected using a two-way analysis of variance, correcting for age, years of education, and head motion.

**Results:**

A significant interaction effect between tobacco addiction and weight status is shown in right superior frontal gyrus. Correlation analyses show that the strengthened ReHo value in the right superior frontal gyrus is positively associated with pack-year. Besides, the main effect of tobacco addiction is specially observed in the occipital lobe and cerebellum posterior lobe. As for the main effect of weight status, the right lentiform nucleus, left postcentral gyrus, and brain regions involved in default mode network (DMN) survived.

**Conclusions:**

These results shed light on an antagonistic interaction on brain ReHo between tobacco addiction and weight status in the right superior frontal gyrus, which may be a clinical neuro-marker of comorbid tobacco addiction and overweight. Our findings may provide a potential target to develop effective treatments for the unique population of comorbid tobacco addiction and overweight people.

## Introduction

Smoking and being overweight could lead to adverse health effects and the associated diseases, such as cardiovascular diseases, type II diabetes, and cancers remain an important public health problem (1). Every year, comorbid tobacco smoking and obesity cause 10 times more deaths than the opioid epidemic (2). Men had a much higher prevalence of ever-smoking and current smoking (67.39 and 48.77%) than women (3.74 and 2.93%) in China (3). Nicotine, a main component of tobacco, is the primary reason for tobacco addiction. Tobacco addiction is a chronic, relapsing mental disorder characterized by impaired inhibitory control and compulsive tobacco seeking and smoking (4). Overweight is most commonly identified using anthropometric measurements, such as body mass index (BMI), which ranges from 25 to 29.9 kg/m^2^ (5). Overweight status may reflect a prodrome to obesity and is associated with an increased risk of premature mortality compared with healthy BMI controls (5). Impulsivity and compulsivity are multidimensional constructs that are increasingly recognized as high weight determinants (6). Together, it has been shown that tobacco addiction and overweight have a close link in both epidemiology and neurobehavior (7–9). Nonetheless, few researchers have directly investigated the interaction between long-term smoking and weight condition at the neurobiological level. Understanding the neural mechanism of comorbid tobacco addiction and overweight may help to facilitate the development of target therapeutic strategies for this special population.

The nature of addiction is frequently considered as either a voluntary behavior or a biological vulnerability (10). Current evidence suggests that tobacco use exerts its initial reinforcement effect by activating the reward network in the cerebrum. This process was debated as a “personal lifestyle choice” (10). Continuously, tobacco abuse impairs brain function by disturbing the ability of self-control, which turns initial voluntary action into the automatic and compulsive behavior that characterizes addiction (10, 11). A meta-analysis found that substance addiction subjects (e.g., tobacco, cocaine, and alcohol) showed decreased gray matter volume in the orbitofrontal cortex (OFC), insula, anterior cingulate (ACC), and striatum (12). A recent study on smoking cessation suggested that repeated tobacco exposure could “hijack” natural reward circuitry by increasing the desire to obtain tobacco, and smoking cessation would restore striatal resting-state functional connectivity (13, 14). Moreover, serious tobacco addiction could affect brain regional spontaneous activity in the higher functional cortex, such as the superior frontal gyrus and precentral gyrus (15). Combined, the reward circuit and executive control system play an important role in the development of tobacco addiction.

Similarly, consuming foods with high in fat and sugar also sensitizes the dopaminergic mesocorticolimbic nervous system (16, 17). Being overweight is related to impair executive functions, such as reward valuation, decision-making, and inhibitory control, which is considered the reason for the difficulties sustaining a healthy diet. Growing evidence demonstrates that such abnormalities are accompanied by disruptions in functional brain networks, particularly those that support reward valuation, self-regulation, and self-control (18, 19). In a food cue–induced functional MRI study, BMI was associated with the increased functional connectivity in fronto-striatal circuits and default mode networks (DMNs) (9). Subjects with high BMI also tended to smaller, immediate rewards rather than larger, delayed rewards comparing with normal weight subjects, which motivated overconsumption of food and lead to excessive weight gain (20, 21). Together, previous studies suggest deficiencies in self-control and reward valuation could impact decisions on diet and lead to overweight.

Growing evidence has suggested that both tobacco addiction and overweight have effect on reward circuit and executive control system (22). Brain functional studies have found altered brain activity in the prefrontal cortex in both the main effects of tobacco addiction (smokers vs. non-smokers) and the main effect of weight status (overweight vs. normal weight), demonstrating an additive effect of tobacco addiction and overweight (15, 23, 24). Moreover, Alice V. Ely's study demonstrated that BMI impacted activation in the right dorsolateral prefrontal cortex (dlPFC) in response to smoking cues, with significantly reduced response in overweight smokers compared with normal-weight smokers (1). However, existing studies have just focused on the influence of tobacco addiction or being overweight on brain activity, respectively. The interaction between the two factors was ignored in this process (4, 24). More important, weight gain is considered one of the main reasons for stopping smoking cessation treatments because the long-term reward neuroadaptations induced by tobacco (25) as well as biological susceptibility may contribute to the rewarding value of highly palatable food in the absence of tobacco (22). Therefore, assessing the interaction effects and neural mechanism of the tobacco addiction-overweight comorbidity is quite necessary.

Regional homogeneity (ReHo) is a relatively new method for measuring local resting functional connectivity, which has been demonstrated a promising biomarker in various mental disorders (23, 26–28). ReHo is a voxel-based method to estimate cerebral activity of the given voxel and its closest neighbors by means of measuring similarity or synchronicity between time series using Kendall's coefficients (29). Several researchers have demonstrated that the ReHo value could reflect the difference between individuals with substance addiction and healthy controls (23, 30, 31). Chen's study found decreased ReHo value in right superior frontal gyrus, bilateral precuneus, and bilateral middle cingulum gyrus among long-term smokers compared with healthy controls (23). To sum up, it has been shown that ReHo is relatively efficient both theoretically and practically. Besides, the ReHo value in subjects with tobacco addiction-overweight comorbidity has not yet been quantified.

In this study, we recruited four groups, such as overweight smokers, normal-weight smokers, overweight healthy controls, and normal-weight healthy controls. The aim of our study was that (i) estimate whole brain ReHo value to measure intrinsic brain activity and explore whether an interaction exists between tobacco addiction and weight status. (ii) Assess whether tobacco addiction diagnosis and weight status affected certain brain regions. (iii) Conduct correlation analysis to explore the links between such affected brain areas and the degree of tobacco addiction and overweight. In this current study, we assumed that the interaction between tobacco addiction and weight status would show altered brain activity in reward network and executive control network, and such changes would be associated with the degree of tobacco addiction and overweight.

## Methods and Materials

### Participants

The potential participants were men from the local community recruited through advertisements. A total of 89 male subjects (aged 20–55 years) were recruited for this study, including four groups: (i) overweight long-term smokers (*n* = 24); (ii) normal-weight smokers (*n* = 28); (iii) overweight non-smokers (*n* = 19), and (iv) normal-weight non-smokers (*n* = 28). Long-term smokers were defined as individuals who smoked at least 10 cigarettes daily in the past 2 years, and met the DSM-V criteria for tobacco use disorder, and had no period of smoking abstinence longer than 3 months (4, 10). We used Fagerström Test for Nicotine Dependence (FTND) to measure the severity of tobacco addiction and collected clinical information related to smoking, such as smoking on set, duration, and cigarettes per day (32). Non-smokers were included subjects who smoked less than five cigarettes in their lifetime (33). According to their BMI, participants were separated into a normal weight group (BMI < 25.0) and overweight group (BMI arrange 25–29.9) (34). The exclusion criteria for all participants were as follows: (i) existence of neuropsychiatric diseases; (ii) systemic diseases (such as, diabetes, hypertension, and cerebrovascular disease); (iii) current use of psychotropic medications or concurrent substance abuse, such as alcohol and heroin; (iv) evidence indicating that overweight differs substantially from obesity and the two weight conditions have different effects on brain activity (34).

Therefore, this study focused on overweight status and excluded obesity individuals; (v) because the rates of smoking in Chinese men are much higher and the public health burden falls predominately on this group. The current study focused on male smokers and excluded female smokers (3); or (vi) claustrophobia and other contraindications to magnetic resonance imaging (MRI). The experiment was approved by the Medical Ethics Committee of First Affiliated Hospital of Zhengzhou University, and informed consent was obtained from each participant.

### Image Acquisition

At the First Affiliated Hospital of Zhengzhou University, MRI data were obtained using a 3.0T German Siemens Magnetom Skyra MRI equipment with a sixteen-channel prototype quadrature birdcage head coil. Participants were instructed to rest with their eyes closed, keeping awake, not to think of anything, and to keep their head motionless during scanning. Earplugs were used to protect the hearing of subjects, and spongy pads were used to fix their heads to minimize head movement. No external stimuli were exerted during image acquisition. Resting-state functional images were collected using an echo-planar imaging sequence. The parameters were repetition time (TR)/echo time (TE) = 2,000/30 ms, flip angle = 80 degrees, matrix size = 64 × 64, field of view = 240 × 240 mm, voxel size = 3.4 × 3.4 × 4 mm, slices = 36, and slice thickness = 4 mm, a total of 180 volumes. All slices along the AC-PC line were acquired with a total scan time of 360 s.

### Data Analyses

The Data Processing and Analysis of Brain Imaging (DPABI v3.0) (http://rfmir.org/dpabi) toolbox was used to preprocess the functional imaging data. Preprocessing comprised format conversion (DICOM to NIFTI), discarding the first 5 volumes, slice-timing, and realignment (cut off < 2.5 mm or 2.5 degrees). No subjects were excluded in this step. The functional images were spatially normalized to the standard Montreal Neurological institute (MNI) template and resampled to 3 × 3 × 3 mm. Subsequently, to remove any residual effects of motion and other non-neuronal factors, nuisance covariates, such as 24 head motion parameters and signals of global signals, white matter, and cerebrospinal fluid were regressed out. Then, linear detrending and temporal band-pass filtering (0.01–0.08 Hz) were performed to remove low- and high-frequency noise. Finally, scrubbing further eliminated the influence of head motion and noise.

The ReHo value was calculated to measure the similarity of the time series of a given voxel to its nearest 27 voxels (29). The Kendall's coefficient of concordance (KCC) value of each voxel was calculated to acquire an individual KCC map or ReHo map. To avoid the impacts of individual variations, whole-brain equalization was performed to normalize ReHo maps for further statistical analysis. Finally, the ReHo maps were smoothed with a Gaussian kernel of full-width at half-maximum of 6 mm.

### Statistical Analyses

Demographic and clinical data were analyzed using a two-sample *T*-test. Differences were considered significant at *p* < 0.05. Using the full factorial model in SPM12, a two-way analysis of variance (ANOVA) was performed for whole brain ReHo comparisons to analyze the interaction effects between the addiction group (smokers and non-smokers) and weight status (normal weight and overweight), with age, years of education, and mean FD as covariates [Gaussian random field theory (GRF) corrected, *p* voxel < 0.005, and *p* cluster < 0.05]. Each identified cluster where the ReHo value was found to be significant for the interact effect of tobacco addiction and weight status was set as the region of interest (ROI). The *post-hoc* analysis was conducted to compare groups difference by two-sample *T*-test and to correct for multiple comparisons (*p* < 0.05/4 for interaction effect analyses and Bonferroni corrected).

### Correlation Analyses

Correlation analyses were conducted to investigate the relationship between the ReHo value alterations in ROI and tobacco addiction severity (pack-year and FTND score) in the two smoking groups, as well as the relationship between ReHo value and BMI in the two overweight groups, separately. We used Spearman's correlation as a more robust measure for ReHo-clinical correlation (35).

## Results

### Demographic and Clinical Data

Long-term smokers and non-smokers included in the study have no significant differences in age, years of education, mean FD, and BMI. Normal weight individuals and overweight individuals have no significant differences in age, years of education, and mean FD, either. Normal weight smokers did not differ from overweight smokers in FTND, smoking onset, and lifetime smoking (pack-year). The detailed demographic information is displayed in Table 1.

**Table 1:** Demographic and clinical characteristics of subjects.

**Demographics**	**Smokers**		**Non-smokers**		**Comparison**	
	**OW-SM (*n* = 24)**	**NW-SM (*n* = 28)**	**OW-noS (*n* = 19)**	**NW-noS (*n* = 28)**	**SM vs. noS**	**OW vs. NW**
Age (year)	31.80 ± 1.16	31.29 ± 1.05	33.05 ± 1.39	31.68 ± 1.24	*t* = −0.596 (*P* = 0.552)	*t* = 0.718 (*P* = 0.474)
Education (year)	15.30 ± 0.32	15.54 ± 0.25	14.56 ± 0.68	16.32 ± 0.61	*t* = −0.395 (*P* = 0.694)	*t* = −1.960 (*P* = 0.053)
Age onset of smoking	18.30 ± 0.67	19.39 ± 0.56	—	—	—	*t* =-1.262 (*P* = 0.213)
Smoking years	13.54 ± 1.17	11.82 ± 1.09	—	—	—	*t* = 1.077 (*P* = 0.287)
Pack-year	14.34 ± 1.68	10.08 ± 1.50	—	—	—	*t* = 1.859 (*P* = 0.064)
Cigarettes/day	20.50 ± 1.61	16.11 ± 1.58	—	—	—	*t* = 1.940 (*P* = 0.058)
FTND	3.83 ± 0.47	3.54 ± 0.38	—	—	—	*t* = 0.494 (*P* = 0.623)
BMI	27.24 ± 0.39	22.03 ± 0.34	27.00 ± 0.42	22.73 ± 0.67	*t* = −0.038 (*P* = 0.969)	—

*Data represent mean ± SEM; FTND, Fagerström Test for Nicotine Dependence; peak-year, years of smoking × cigarettes smoked per day/20; BMI, body mass index; OW-SM, overweight smokers; NW-SM, normal-weight smokers; OW-noS, overweight non-smokers; and NW-noS, normal-weight non-smokers*.

### Interaction Effects

**Figure 1:**
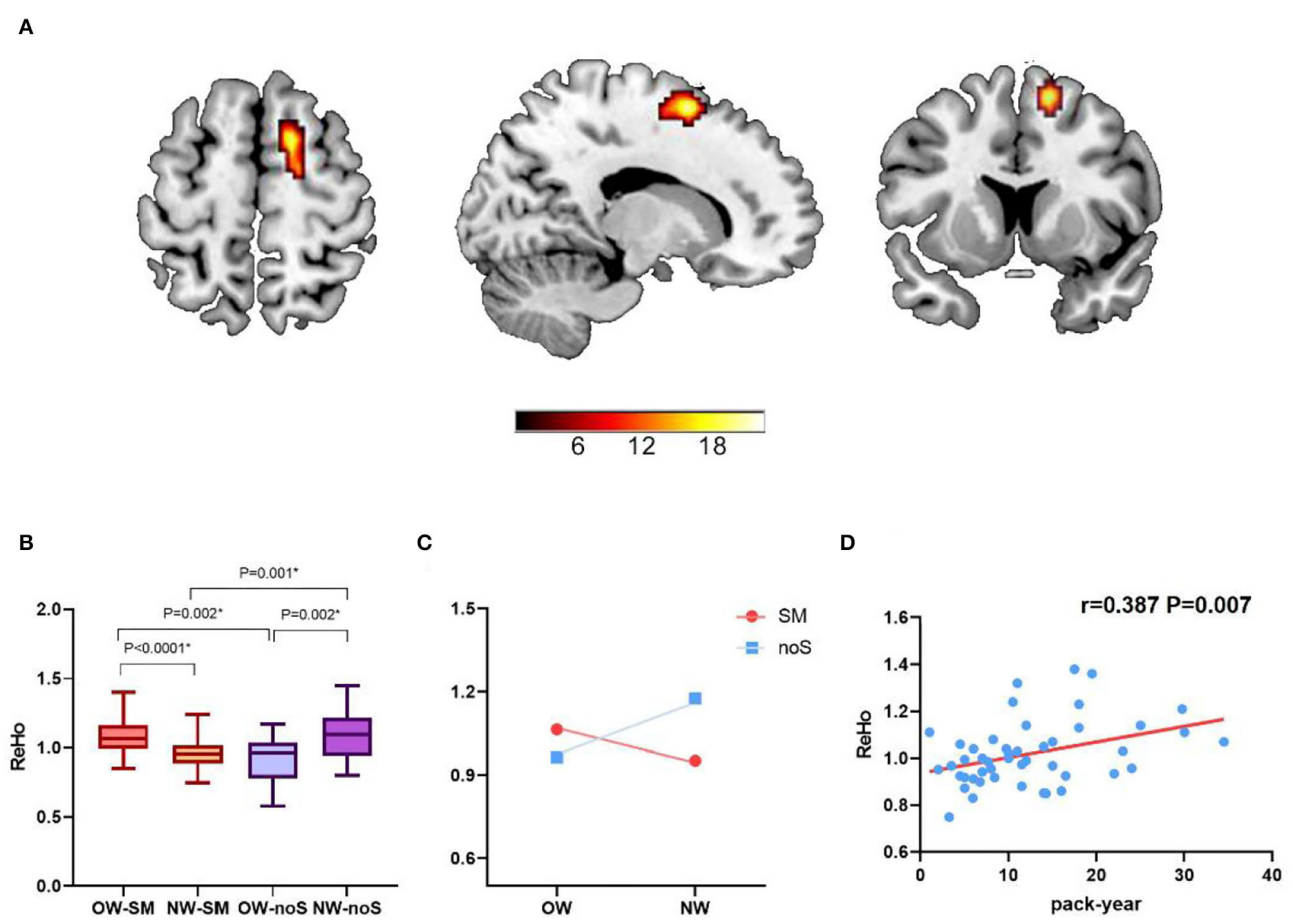
The interaction effect of tobacco addiction and weight status. **(A)** A significant interaction effect shown by regional homogeneity (ReHo) in right superior frontal gyrus using two-way ANOVA (Gaussian random field theory (GRF) corrected, *p* voxel < 0.005, and *p* cluster < 0.05). **(B,C)** Planned *post-hoc* analysis of the right superior frontal gyrus among the four groups. The vertical bar indicates the maximum and minimum across subjects. *p < 0.05/4, Bonferroni corrected. **(D)** The ReHo value in right superior frontal gyrus was positively correlated with pack-year (*r* = 0.387, *p* = 0.007, and Bonferroni corrected).

An interaction effect of tobacco addiction × overweight is showed in the right superior frontal gyrus (peak MNI: 15, 9, 60; cluster size: 50; and peak F value: 22.31), GRF corrected, *p* voxel < 0.005, and *p* cluster < 0.05. Planned *post-hoc* analysis for the right superior frontal gyrus shows significantly increased ReHo value in overweight long-term smokers compared with normal weight long-term smokers (*t* = 3.768, *p* < 0.0001, and Bonferroni corrected). While the ReHo value in overweight non-smokers is decreased related to normal weight non-smokers (*t* = −3.242, *p* = 0.002, and Bonferroni corrected). Normal weight long-term smokers show decreased ReHo value comparing with normal weight non-smokers (*t* = −3.540, *p* = 0.001, and Bonferroni corrected). Besides, overweight long-term smokers show increased ReHo value comparing with overweight non-smokers (*t* = 3.392, *p* = 0.002, and Bonferroni corrected) (Table 2; Figure 1). Moreover, correlation analysis finds that ReHo value in the right superior frontal gyrus is positively correlated with pack-year (*r* = 0.387, *p* = 0.007, and Bonferroni corrected). No significant linear correlations are found with FTND scores and BMI.

**Table 2:** Significant group differences in regional homogeneity (ReHo).

**Cluster Location**	**Peak (MNI)** **(X, Y, Z)**	**Cluster size**	**Peak F value**
**Interaction effect**			
Superior frontal gyrus R	15, 9, 60	50	22.31
**Main effect (tobacco addiction)**			
Cerebellum posterior lobe R	36, −78, −42	53	15.76
Precentral gyrus R	45, −12, 54	54	16.61
Inferior frontal gyrus L	−51, 42, 15	33	19.35
Lingual gyrus R	24, −78, −6	43	17.75
Fusiform gyrus L	−36, −66, −15	70	21.54
Occipital inferior gyrus L	−30, −78, −9	25	13.81
Calcarine L	−18, −63, 9	83	13.88
**Main effect (weight status)**			
Lentiform nucleus R	21, −15, 3	31	16.60
PCC	3, −51, 12	48	19.59
Postcentral gyrus L	−51, −9, 15	32	18.17
Superior frontal gyrus L	−18, 51, 33	46	13.74
Inferior parietal lobule L	−60, −42, 45	45	13.64

*PCC, posterior cingulate cortex; MNI, Montreal Neurological Institute; R, right; L, left*.

### Main Effects

**Figure 2:**
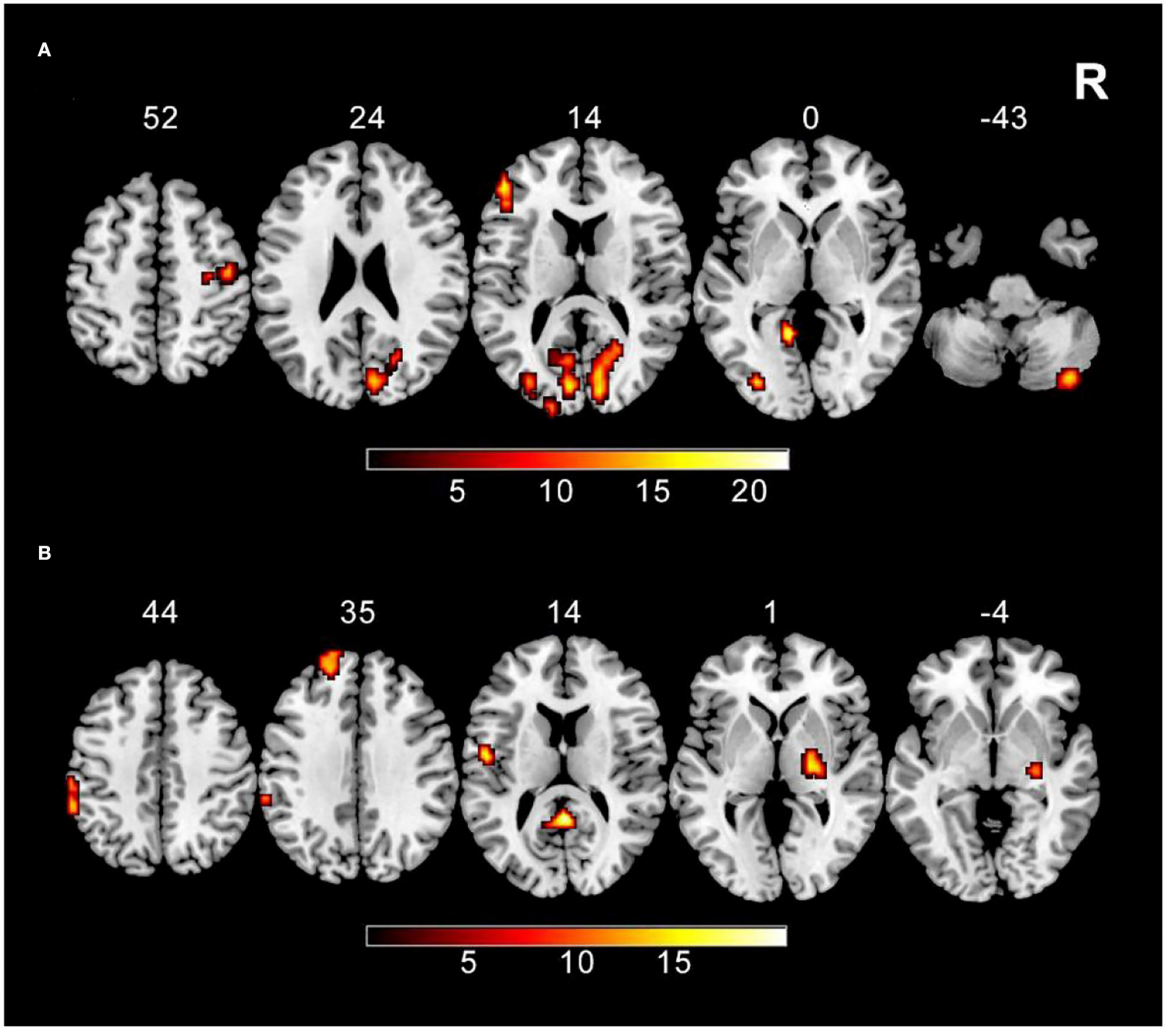
The main effect results **(A)** ReHo shows the significant main effect of tobacco addiction in the right cerebellum posterior lobe, right precentral gyrus, left inferior frontal gyrus, right lingual gyrus, left fusiform gyrus, left occipital inferior gyrus, and left calcarine using two-way ANOVA (GRF corrected, *p* voxel < 0.005, and *p* cluster < 0.05). **(B)** ReHo shows the significant main effect of weight status in the right lentiform nucleus, posterior cingulate, left postcentral gyrus, left superior frontal gyrus, and left inferior parietal lobule using two-way ANOVA (GRF corrected, *p* voxel < 0.005, and *p* cluster < 0.05).

The main effect of tobacco addiction is showed in the right cerebellum posterior lobe, right precentral gyrus, left inferior frontal gyrus, right lingual gyrus, left fusiform gyrus, left occipital inferior gyrus, and left calcarine (GRF corrected, *p* voxel < 0.005, and *p* cluster < 0.05). As for the main effect of weight status, right lentiform nucleus, posterior cingulate, left postcentral gyrus, left superior frontal gyrus, and left inferior parietal lobule are survived (GRF corrected, *p* voxel < 0.005, and *p* cluster < 0.05) (Table 2; Figure 2).

## Discussion

This study focused on the interaction between tobacco addiction and weight status by means of a 2 × 2 factorial design. ReHo, a relatively new method, was used to measure local resting functional connectivity, reflecting different brain functional activity among four groups. Long-term smokers compared with healthy controls displayed abnormal ReHo, especially in the occipital lobe and cerebellum posterior lobe, irrespective of weight status. Overweight individuals showed aberrant ReHo in the right lentiform nucleus, left postcentral gyrus, and brain regions involved in DMN compared with normal weight individuals. More importantly, planned *post-hoc* analysis showed that the combined effects of tobacco addiction and overweight were less than the sum of the two factors separately, which uncovered an antagonistic interaction on ReHo in the right superior frontal gyrus between tobacco addiction and weight status, and such alteration was positively correlated with pack-year.

The antagonistic interactions between tobacco addiction and weight status on ReHo of the right superior frontal gyrus are in line with the hypotheses of neuronal overlaps on reward-related brain regions between the two factors (22). Previous studies have reported many interesting findings, such as weight gain and increased appetite after tobacco quitting, nicotine leading to anorexia, and smokers are usually leaner and have higher basal metabolic rate (BMR) than non-smokers (36–41). Such findings may suggest the interaction relationship between smoking and weight status, and our results highlight the neurological mechanism to explain this phenomenon. The superior frontal gyrus, located in the upper prefrontal cortex, has been reported to be involved in cognition and motor control, working memory, and decision-making processes that were broadly impacted by the dopaminergic responses to reward evaluation (42). Individuals with food addiction showed increased activation in the superior frontal gyrus in response to the highly processed food cues comparing with healthy controls (43). Besides, the superior frontal gyrus was considered as a possible target of neuromodulation in overweight/obesity for it can significantly predicted BMI (44, 45). Similarly, in heavy smokers, spontaneous activity in the superior frontal gyrus was also affected by chronic tobacco intaking (15). Combined, we infer that the superior frontal gyrus is the shared neurobiological substrate in tobacco addiction and overweight. Besides, *post-hoc* analysis of the right superior frontal gyrus characterized antagonistic interaction between the two factors. Previous studies suggested that the superior frontal gyrus was anatomically connected with other regions in prefrontal cortex through arcuate fibers, and was considered as a connection node between central executive network (CEN) and DMN, which played an important role in allocating attention efficiently (46). A recent resting-state fMRI study has indicated that overweight could dampen the effect of smoking on DMN-ECN circuit, and further contribute to the circuit dysfunction (34). In line with these studies, our research found brain functional abnormalities in superior frontal gyrus, which might impact the dynamic interaction between brain networks and further lead to a failure to modulate attention and behavior. Such abnormalities may explain the smoking cessation difficulties in comorbid tobacco addiction and overweight individuals (47, 48). Moreover, correlation analysis found that more serious tobacco addicts showed increased ReHo in superior frontal gyrus, but there was no significant linear correlation with BMI. The non-linear relationships (quadratic effect) between resting state functional connectivity and BMI reported in Alice V. Ely's study suggested the non-monotonic feature of brain activity (1).

In addition, we noted that the factor of tobacco addiction impacted the ReHo value especially in the inferior frontal gyrus, occipital lobe, and cerebellum posterior lobe. The occipital lobe, such as lingual gyrus, fusiform gyrus, is the visual cortex involved in processing color information, face and body recognition, and emotion perception responding to facial stimuli (49–51). A large scale meta-analysis has demonstrated that individuals with substance addiction, such as tobacco, alcohol, and cocaine displayed higher gray matter volume in the right lingual gyrus and right fusiform gyrus relative to healthy controls (12). Previous studies observed the brain regions associated with visuospatial attention cortex (prefrontal cortex and fusiform gyrus) were much more active after exposure to smoking-related images than neutral images in long-term smokers (52). These are consistent with our findings and reflects the deficits in visuospatial attention in long-term smokers. As for cerebellum, growing evidence indicated that it was connected to cerebral cortex in both anatomy and function and played an important role in regulating emotion and decision-making (53, 54). Our results supplement previous findings and highlight the non-motor function of cerebellum.

As for the factor of weight status, we found that overweight individuals showed abnormalities in brain regions that were mainly concentrated in the right lentiform nucleus, left postcentral gyrus, and brain regions involved in DMN comparing with normal weight individuals. DMN implicated in interoceptive awareness and mental imagery, and is considered as a “cohesive connector” system in resting-state, integrating information between- and within-network (55, 56). Our findings show that higher BMI alters the internal cohesiveness in DMN, affecting efficient processing of internal functions among overweight subjects, such as the procedure of the food and non-food related reward (57). Furthermore, lentiform nucleus, the vital neural nucleus in basal ganglia that includes globus pallidus and putamen, which is thought to be part of the reward system and has an effect on reward processing and motivation (58, 59). In our research, the functional alterations of lentiform nucleus and DMN in overweight individuals may support the hypothesis of “food addiction”—exposure to food alters brain reward circuits, touching off addiction-like behavioral phenotype of compulsive overeating, just like tobacco addiction (60–62).

There are a few limitations in our study. First, though our study sheds new light on the neuro-biologic interactions of tobacco addiction and weight status, the causality between cerebral activity and development of addiction-related disorders remains unexplainable according to cross-sectional study. Second, as for the factor of weight status, we only recruited overweight individuals and ignored obese individuals (BMI > 30). The relationships between obesity and tobacco addiction need to be further explored. Third, the sample size is small and the subjects recruited in this study were male adults, while women were not included, so the results of this study are not applicable to all population (63).

## Conclusion

Overall, our findings shed light on an interaction relationship between tobacco addiction and overweight in terms of system-level neurobiological mechanism. Specifically, findings revealed an antagonistic interaction on the local resting functional connectivity in the right superior frontal gyrus between the two factors. Such interaction maybe a clinical neuro-marker of comorbid tobacco addiction and overweight. Future efforts are being undertaken to develop effective treatments that target therapeutic strategies for the special population of comorbid tobacco addiction and overweight. In addition, these current findings also emphasize the importance of controlling another variable when explore the effects of substance addiction or overweight separately in future studies.

## Data Availability Statement

The original contributions presented in the study are included in the article/supplementary files, further inquiries can be directed to the corresponding author/s.

## Ethics Statement

The experiment was approved by the Medical Ethics Committee of First Affiliated Hospital of Zhengzhou University, and informed consent was obtained from each participant. The patients/participants provided their written informed consent to participate in this study.

## Author Contributions

MZ and XG conceived and designed the study, analyzed the data, and drafted the manuscript. ZY, XN, and JChen performed the statistical analyses. SH and YW revised the manuscript. YZ supervised. YZ, JCheng, and WW provided financial support. All authors take responsibility for the content, gave approval of the submission and made substantive intellectual contributions to the submitted work.

## Funding

This work was supported by Medical Science and Technology Research Project of Henan Province, Grant/Award Number: 201701011; the Natural Science Foundation of China, Grant/Award Numbers: 81871327 and 81601467.

## Conflict of Interest

The authors declare no potential conflicts of interest.

## Publisher's Note

All claims expressed in this article are solely those of the authors and do not necessarily represent those of their affiliated organizations, or those of the publisher, the editors and the reviewers. Any product that may be evaluated in this article, or claim that may be made by its manufacturer, is not guaranteed or endorsed by the publisher.
